# HBSP inhibits tubular cell pyroptosis and apoptosis, promotes macrophage M2 polarization, and protects LPS‐induced acute kidney injury

**DOI:** 10.1111/jcmm.70202

**Published:** 2024-11-25

**Authors:** Lili Huang, Yuanyuan Wu, Wenli Sai, Yanan Wang, Guijuan Feng, Yuqing Lu, Fei Chen, Xinzhong Huang, Hongsheng Zhao, Zhifeng Gu, Bin Yang

**Affiliations:** ^1^ Nantong‐Leicester Joint Institute of Kidney Science Affiliated Hospital of Nantong University Nantong China; ^2^ Department of Critical Care Medicine Affiliated Hospital of Nantong University Nantong China; ^3^ Department of Pathology, Medical School Nantong University Nantong China; ^4^ Clinical Medical Research Center Affiliated Hospital of Nantong University Nantong China; ^5^ Department of Nephrology Affiliated Hospital of Nantong University Nantong China; ^6^ Department of Stomatology Affiliated Hospital of Nantong University Nantong China; ^7^ Department of Rheumatology, Affiliated Hospital of Nantong University, Medical School of Nantong University Nantong University Nantong China; ^8^ Department of Cardiovascular Sciences, College of Life Sciences, University Hospitals of Leicester NHS Trust University of Leicester Leicester UK

**Keywords:** apoptosis, helix B surface peptide, macrophage polarization, pyroptosis, sepsis‐associated acute kidney injury

## Abstract

Sepsis‐associated acute kidney injury (AKI) has high morbidity and mortality, but without cause‐specific treatment. Erythropoietin derived Helix B surface peptide (HBSP) alleviates AKI, whereas its underlying mechanisms remain to be further explored. Here, the effects of HBSP on pyroptosis, apoptosis, macrophage polarization and repair were investigated in lipopolysaccharide (LPS)‐induced AKI mouse model and cultured kidney epithelial cells. Systemic inflammation, compromised renal function and histology were demonstrated in LPS‐treated mice, with upregulated pyroptotic and apoptotic key proteins in the kidneys including GSDMD‐N, cleaved IL‐1β, IL‐18 and caspase‐3. These proteins were localized in tubular areas and colocalized with aquaporin‐1 (AQP1), with increased F4/80^+^ M1 macrophages. However, HBSP mitigated pyroptosis, apoptosis and inflammation, and promoted macrophage M2 polarization. In addition, HMGB1 and erythropoietin receptor (EPOR) were increased by LPS and decreased by HBSP, both of which were positively correlated with pyroptotic and apoptotic proteins. Moreover, HBSP reduced TNF‐α and IL‐6 mRNA levels, as well as pyroptosis and apoptosis in LPS‐stimulated TCMK‐1 cells. In conclusion, HBSP inhibited tubular pyroptosis and apoptosis, EPOR expression, promoted macrophage M2 polarization, and protected against LPS‐induced AKI. These findings provide new mechanistic insights into the renoprotection of HBSP, and facilitate its potential for clinical applications and therapeutic strategies in sepsis‐associated AKI.

## 1 INTRODUCTION

Sepsis‐associated acute kidney injury (SA‐AKI) is a common and severe complication in critically ill patients, leading to the increased risk of mortality. The incidence of SA‐AKI among critical patients is approximately 50%,^1^, ^2^ and the mortality is in a range of 11% to 77%,^3^ which is significantly higher than that of patients without sepsis‐AKI.^4^ Even with timely diagnosis, there is no cause‐specific treatment at present, while available supportive and replacement treatment have not fundamentally improved the outcome of SA‐AKI.^5^, ^6^, ^7^ The pathogenic mechanisms of SA‐AKI are multifaceted and complicated, which have not yet fully elucidated.

Renal tubular epithelial cells (TECs) play central roles in the development of SA‐AKI.^8^ Lipopolysaccharide (LPS), a well‐known circulating endotoxin, is one of major causes of sepsis, and also an inducer of programmed TEC death in SA‐AKI.^9^ Caspase‐3 is an executor of apoptosis, and silencing caspase‐3 significantly improved ischemia–reperfusion (IR) injury in transplanted auto kidneys of porcine models,^10^ while caspase‐3, as a mediator of inflammation, also played key roles in SA‐AKI.^11^ In addition, TEC pyroptosis provokes the release of pro‐inflammatory cytokines and damage‐associated molecular patterns (DAMPs) including high mobility group box 1 (HMGB1) at the early stage of LPS‐induced AKI.^12^, ^13^ Pyroptosis is mediated by the activation of gasdermin domain (GSDMD), which also contributed to cisplatin and IR‐induced AKI, but in GSDMD^−/−^ mice renal function, inflammatory cytokine secretion and tubule cell pyroptosis were alleviated.^14^, ^15^ DAMPs released by pyroptotic TECs promoted the transformation of macrophages to the pro‐inflammatory phenotype M1, leading to aggravated tissue damage, inflammation and subsequent fibrosis. Furthermore, apoptotic cells and certain inflammatory factors can also initiate macrophage polarization towards the anti‐inflammatory phenotype of M2, thereby mediating renal regeneration and repair.^16^ Previous study elucidated that M1 macrophages aggravated the pyroptosis of TECs, whereases M2 macrophages ameliorated TEC injury.^17^ Reducing macrophage infiltration was beneficial to improve the outcome of SA‐AKI.^18^ The regulation of TEC death and macrophage infiltration and polarization are strategic approaches for the therapeutic intervention of LPS‐induced AKI.

Helix B surface peptide (HBSP), derived from erythropoietin (EPO), consists of 11 amino acids and selectively binds to the innate repair receptor of EPOR and β common receptor (EPOR/βcR), but not homodimer (EPOR)_2_, thus mediating tissue protection without erythropoiesis.^19^ It was shown that HBSP preserved kidney structure and function by modulating cell death and immune responses in renal IR injury models.^20^, ^21^ The expressed EPOR and EPOR/βcR were located on TECs and upregulated by kidney IR injury at the acute stage, which prime the protective role of HBSP.^21^, ^22^ In addition, EPOR/βcR expressed on the cellular membrane of macrophages mediated the reduction of secreted pro‐inflammatory cytokines under TNF‐α induced cellular stress *in vitro*.^23^ EPOR was also mediated the internalization of apoptotic cells to promote immune tolerance, which may attribute to the heterodimer receptor EPOR/βcR.^24^ Moreover, HBSP exhibited anti‐inflammatory effect by inhibiting NF‐κB activity in LPS‐stimulated primary macrophages.^25^ HBSP reduced systemic inflammation and preserved kidney function both in LPS and IR‐induced AKI via PI3K/Akt Pathway *in vivo*.^24^, ^26^ However, the exact therapeutic impacts and mechanisms of HBSP in SA‐AKI need be further defined.

This study showed that HBSP treatment significantly improved systemic inflammation, kidney functional and structural damage, and macrophage M1 polarization, triggered by LPS. The renoprotection of HBSP might be due to modulating the pyroptotic and apoptotic cell death of TECs, as well as the immune response of macrophages. Our study provided the additional evidence of renoprotection induced by HBSP and its underlying mechanisms in LPS‐induced AKI and its associated models.

## 2 MATERIALS AND METHODS

### 2.1 LPS‐induced AKI mouse model

Male C57BL/6 mice, 8–10 weeks, provided by the Jiangsu Huachuang Xinnuo Pharmaceutical Technology Co., Ltd., Taizhou, China. All animal procedures were performed in accordance with the guidelines of the Animal Care and Use Committee of Nantong University and the Laboratory Animal Monitoring Committee of Jiangsu Province. The animals were housed in an environment with 12‐h light/dark cycles, constant 25°C and 55% humidity, free access to standard laboratory mouse chow and water. The mice were randomly divided into the following 3 groups: Control (*n* = 6), LPS (*n* = 8) and LPS + HBSP (*n* = 8). LPS extracted from Escherichia coli 055: B5 (L2880, Sigma‐Aldrich, St. Louis, USA) was administrated via intraperitoneal injection at a dose of 20 mg/kg body weight. In addition, 24 nmol/kg HBSP (PO16040905, Shanghai Science Peptide Biological Technology, Shanghai, China) was injected intraperitoneally right after the injection of LPS. The mice in the control group were treated with the same volume of saline (*n* = 6). Post‐treatment for 24 h, the animals were anaesthetised with the inhalation of 2.5% isoflurane (R620‐S1, RWD Life Science, Shenzhen, China) in oxygen and the whole blood was collected via cardiac puncture and kidneys were then harvested for further analysis.

### 2.2 Enzyme‐linked immunosorbent assay (ELISA)

The level of IL‐6 in the serum were measured with an ELISA kit (M6000B, R&D Systems, Minneapolis, USA) following the instruction of manufacturer. The optical density value was detected at 450 nm on a microplate reader (Thermo scientific).

### 2.3 Renal function

The level of serum creatinine (SCr) and blood urea nitrogen (BUN) was measured using a QuantiChrom™ Creatinine Assay Kit (BioAssay Systems, Hayward, USA) and a Urea Nitrogen/Urea Content Assay Kit (BC1535, Solarbio, Beijing, China) according to the instruction of manufacturer, respectively.

### 2.4 Histology

Briefly, kidney tissues were fixed in 10% formalin overnight, subsequently dehydrated, embedded in paraffin and sliced to 3.5 μm sections. The tissue sections were then stained with haematoxylin and eosin to assess the score of tubulointerstitial damage (TID) including vacuolation and TEC sloughing off, interstitial expansion and tubular dilation referred according to our previous studies.^27^ The assessment of 20 cortical fields per kidney at 400× magnification was conducted by two researchers independently and blinded to the coding of sections. The average of the scores from 20 fields per animal was used for statistical analysis.

### 2.5 Immunohistochemistry (IHC) staining

Paraffin‐embedded renal sections were dewaxed and antigen retrieved in EDTA antigen retrieval solution (E673003, BBI, Shanghai, China) for 10 min using a high‐pressure steam cooker. Sections were then blocked with QuickBlock™ blocking buffer (PV6000, ZSGB‐BIO, Beijing, China) for 10 min at room temperature for 30 min. Primary antibodies including rabbit anti‐F4/80 (28463‐1‐AP, Proteintech, Chicago, USA), mouse anti‐caspase‐3 (66470‐2‐Ig, Proteintech) and rabbit anti‐EPOR (PAB18350, Abnova, Taibei) were applied and incubated overnight at 4°C. For the negative control, normal mouse/rabbit IgG (2729S, CST, Danvers, USA) was used at the same protein concentration of the primary antibody. The next day, the secondary anti‐mouse/rabbit IgG antibody (PV‐6000, ZSGB‐BIO) was applied to the sections for 20 min at room temperature. The detection of antibody binding was performed with 3,3′‐diaminobenzidine (DAB, Vector, Burlingame, USA) or 3‐amino‐9‐ethylcarbazole (AEC, Vector), followed by counterstaining with haematoxylin. Positively stained areas were assessed at 400× magnification in 20 fields per section by two independent researchers blinded to the coding of sections.

### 2.6 Immunofluorescence staining

For immunofluorescence staining, 10 μm cryosections were prepared from OCT embedded mouse kidney tissues. After rewarming, fixation and antigen retrieval, the sections were blocked using 10% goat serum for 20 min at room temperature. Primary antibodies (Proteintech) were then applied overnight at 4°C including mouse anti‐AQP1 (66805‐1‐Ig), rabbit anti‐NLRP3 (19771‐1‐AP), rabbit anti‐IL‐18 (10663‐1‐AP), rabbit anti‐IL‐1β (16806‐1‐AP) and rabbit anti‐GSDMD‐N (ab215,203, Abcam, Cambridge, UK). After rinsing, secondary antibody (Abcam, CA, USA) including Alexa Fluor®647 goat anti‐mouse (ab150115), Alexa Fluor®647 goat anti‐rabbit (ab150079), Alexa Fluor® 488 goat anti‐rabbit (ab150077) and Alexa Fluor® 488 goat anti‐mouse (ab150113) was applied to the slides at room temperature for 2 h. Nuclei were visualized by counterstaining with DAPI (228,549, Abcam). The staining was viewed under a Fluorescence microscope.

### 2.7 *In situ* end labelling of apoptotic cells

Apoptotic cells within the renal cortex were labelled & detected *in situ* using terminal deoxynucleotidyl transferase‐mediated dUTP nick‐end labelling (S7100, Merck Millipore, Darmstadt, Germany). Positive staining in 20 fields at 400× magnification within the cortex of kidney was assessed by two researchers independently and blinded to the coding of sections.

### 2.8 Flow cytometry

Fresh renal tissues were enzymatically dissociated using 1 mg/mL type IV collagenase and 100 U/mL DNase I (C5138‐5G, Sigma‐Aldrich) for 30 min at 37°C, yielding single‐cell suspensions. Prior to staining, cells were treated with 2.5 μg/mL Fc blocking solution (BD 553141, Biosciences, San Jose, USA) to prevent nonspecific binding. Subsequently, cells were labelled with the following directly conjugated mouse‐specific monoclonal antibodies (BD Biosciences, CA, USA) including PE anti‐CD86 (553692), Alexa Fluor®647 anti‐CD206 (565250), BV421 anti‐F4/80 (565411), APC‐Cy™7 anti‐CD45 (557659) and PerCP‐Cy™5.5 anti‐CD11b (550993). TCMK‐1 cells were stained with Annexin V (AV) and propidium iodide (PI) using an AV‐FITC Apoptosis Detection Kit (BMS500FIC1–300, eBioscience, Vienna, Austria). The stained cells were assessed using a BD FACSCalibur™ Flow Cytometer (BD Biosciences), and the data were analysed using FlowJo™ Software (FlowJo, Ashland, USA).

### 2.9 Cell culture and treatment

TCMK‐1 cells (CCL‐139, American Type Culture Collection, Manassas, USA) were cultured in Dulbecco's modified Eagle's medium (DMEM)/F‐12 medium (Gibco, Carlsbad, USA) supplemented with 10% fetal bovine serum, 100 U/mL penicillin G, and 100 mg/mL streptomycin (Sigma, Dorset, UK). These conditions were sustained within a humidified 5% CO_2_/95% air atmosphere at 37°C. A sepsis associated AKI model *in vitro* was induced by LPS (L6529, Sigma‐Aldrich, St. Louis, USA) at a concentration of 5 μg/mL in TCMK‐1 cells for 24 h, with or without additional HBSP at different concentrations of 5, 10, 20, 40 and 80 ng/mL.

### 2.10 Cell counting kit‐8 (CCK‐8) assay

Cell viability was measured using a CCK‐8 assay kit (C0042, Beyotime, Shanghai, China). The assay was conducted in accordance with the protocol of manufacturer. The absorbance of the samples was measured at 450 nm using a microplate reader (Thermo scientific).

### 2.11 Western blot analysis

Renal tissues and TCMK‐1 cells were lysed using RIPA lysis buffer (P0013B, Beyotime, China). The concentration of lysis was measured with a Pierce™ BCA Protein Assay Kit (23,225, Thermo Fisher scientific, Rockford, USA). Twenty‐five micrograms of supernatant were separated by sodium dodecyl sulfate polyacrylamide gel electrophoresis (SDS‐PAGE) gels and transferred to the polyvinylidene difluoride membranes (03010040001, Roche Diagnostics GmbH, Mannheim, Germany). Followed by blocking for 2 h with 5% milk at room temperature, primary antibodies were applied including mouse anti‐caspase‐3 (1:1000, 66470‐2‐Ig, Proteintech), rabbit anti‐HMGB‐1 (1:5000, ab79823, Abcam), rabbit anti‐GSDMD (1:1000, ab219800, Abcam), rabbit anti‐IL‐1β (1:1000, 16806‐1‐AP, Proteintech), rabbit anti‐IL‐18 (1:1000, 10663‐1‐AP, Proteintech) and mouse anti‐β‐actin (1:10000, ab6276, Abcam, Cambridge, UK) at 4°C overnight. This was followed by a 2‐h room temperature incubation with goat anti‐mouse or rabbit HRP‐conjugated secondary antibodies (14708/14709, CST) respectively. The detection of the antibody binding was conducted using an enhanced chemiluminescence system (32106, Thermo Fisher Scientific). The expression of target proteins was normalized against β‐actin expression, and presented as a ratio against the control group.

### 2.12 Real‐time quantitative polymerase chain reaction (RT‐qPCR) analysis

Total RNA was extracted from renal tissues and TCMK‐1 cells using Trizol reagent (T9424, Sigma‐Aldrich). RT‐qPCR was conducted to determine the mRNA expression level of IL‐6, TNF‐α, IL‐1β and IL‐18 with AceQ® qPCR SYBR Green Master Mix (Q711‐02, Vazyme Biotech, Nanjing, China), corrected by GAPDH. The sequences of related primers were listed in Table 1.

**TABLE 1:** The sequences of primers for RT‐qPCR.

Genes	Type of Primers	Primer sequences
TNF‐α	Forward	5′‐CGCTGAGGTCAATCTGC‐3′
	Reverse	5′‐GGCTGGGTAGAGAATGGA‐3′
IL‐6	Forward	5′‐ACAGAAGGAGTGGCTAAGGA‐3′
	Reverse	5′‐AGGCATAACGCACTAGGTTT‐3′
IL‐1β	Forward	5′‐CACTACAGGCTCCGAGATGAACAAC‐3′
	Reverse	5′‐TGTCGTTGCTTGGTTCTCCTTGTAC‐3′
IL‐18	Forward	5′‐AGACCTGGAATCAGACAACTTT‐3′
	Reverse	5′‐TCAGTCATATCCTCGAACACAG‐3′
GAPDH	Forward	5′‐GCATGGCCTTCCGTGTTC‐3′
	Reverse	5′‐GATGTCATCATACTTGGCAGGTTT‐3′

### 2.13 Statistical analysis

The data are presented as the mean ± standard error of the mean (SEM). Statistical comparisons were performed using one‐way analysis. These analyses were conducted with SPSS Statistics Standard version 26.0 (IBM, New York, USA) or GraphPad Prism version 9.0 (GraphPad Software Inc., San Diego, USA). Data that follow a normal distribution were analysed using the Pearson correlation coefficient method, while data that do not follow a normal distribution were analysed using the Spearman correlation coefficient method. *p <* 0.05 was considered statistically significant.

## 3 RESULTS

### 3.1 HBSP improved systemic inflammation, renal function and histology

**FIGURE 1:**
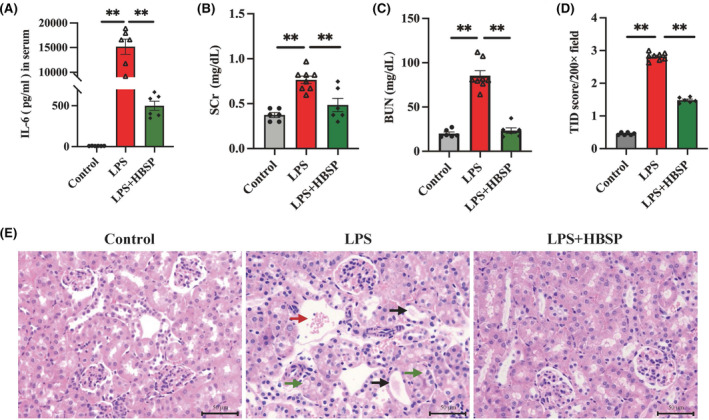
The change of systemic inflammation, renal function and histology in LPS‐AKI mice with or without HBSP treatment. (A) Serum IL‐6 was detected by ELISA. (B, C) Renal function was assessed by SCr and BUN. (D) TID score was semi quantitatively analysed. (E) Representative pictures of histological damage in the cortex of kidneys were shown in haematoxylin and eosin‐stained sections, including interstitial haemorrhage (red arrow), tubular vacuolization (green arrow) and brush border loss and cast formation (black arrow). Scale bar: 50 μm; LPS: 20 mg/kg, HBSP: 24 nmol/kg. The data were presented as mean ± SEM (*n* = 6–8). **p < 0.01; BUN, blood urea nitrogen; ELISA, enzyme‐linked immunosorbent assay; SCr, serum creatinine; TID, tubular interstitial damage.

The serum level of IL‐6 was used to evaluate the systemic inflammation induced by LPS with or without treatment of HBSP, as well as their effects on renal function and structure *in vivo*. Compared to the control group, the level of IL‐6 was significantly increased by LPS, but decreased by HBSP (Figure 1A), which was also applied to the levels of SCr and BUN (Figure 1B,C). The kidney structural damage was revealed by tubular vacuolization, brush border loss and interstitial haemorrhage after LPS induction, which significantly increased the score of TID. However, the score of TID was significantly decreased by HBSP (Figure 1D,E).

### 3.2 HBSP reduced pyroptosis‐associated proteins and inflammation in mouse kidneys

**FIGURE 2:**
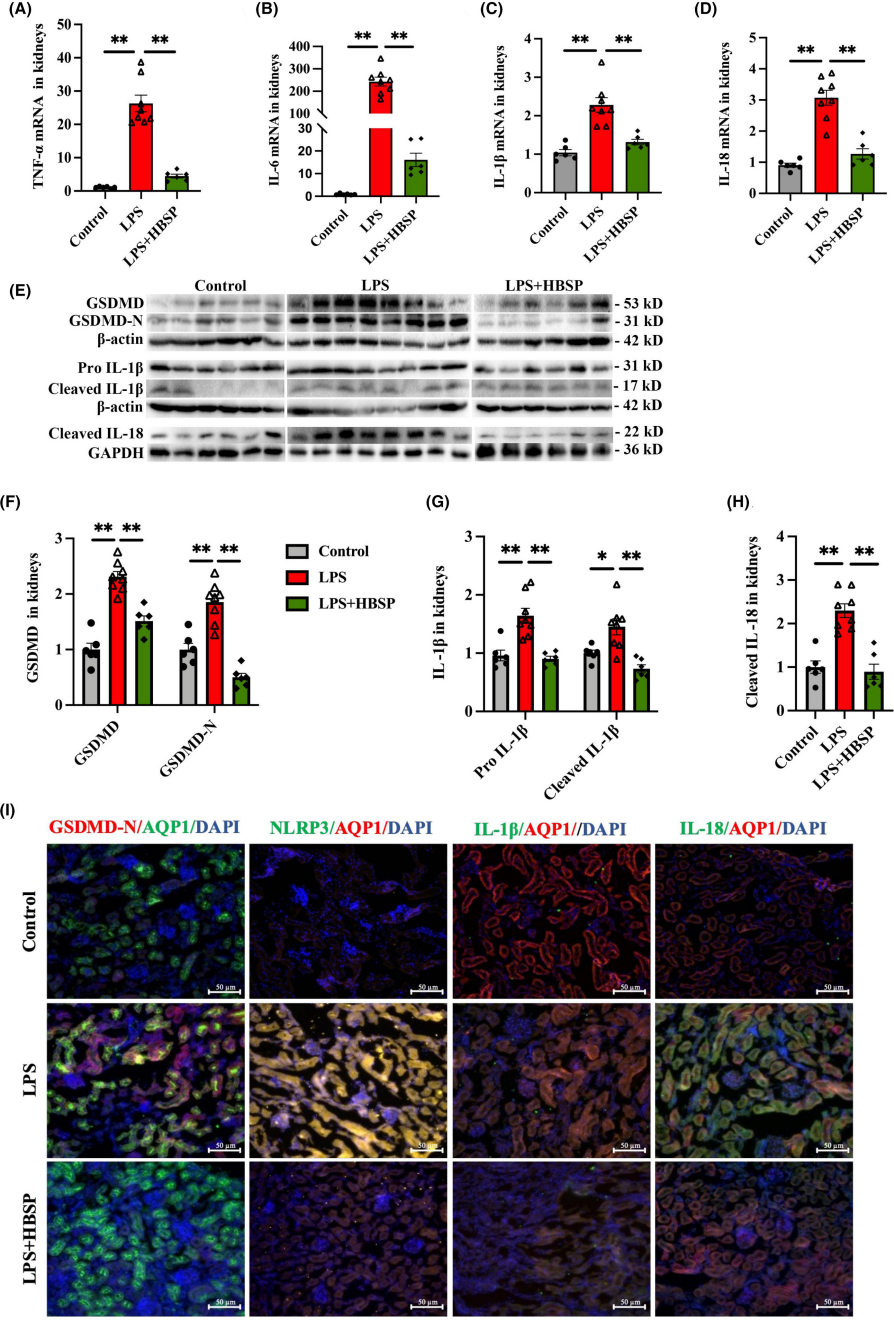
Effects of HBSP treatment on pyroptosis and inflammation in LPS‐AKI mouse kidneys. (A–D) The expression of TNF‐α, IL‐6, IL‐1β and IL‐18 mRNA in kidneys was analysed by RT‐qPCR. (E–H) The expression of pyroptosis‐related proteins was detected by western blotting, including full‐length 53 kD GSDMD and active unit 31 kD GSDMD‐N, full‐length 31 kD and active unit 17 kD IL‐1β, and cleaved 22 kD IL‐18 in kidney homogenates. (I) The co‐localization of pyroptosis‐associated proteins including GSDMD‐N, NLRP3, IL‐1β and IL‐18 with AQP1 (a marker of TECs) in kidneys was detected by immunofluorescence staining. Different colours of fluorescent dyes labelled GSDMD‐N, NLRP3, IL‐1β, IL‐18 and AQP1 respectively. Scale bar: 50 μm. The data are presented as mean ± SEM (*n* = 6–8). **p < 0.01; RT‐qPCR, real‐time quantitative polymerase chain reaction; TECs, tubular epithelial cells.

Pyroptotic cell death was related to kidney damage and repair. As known, pyroptosis is often followed by releasing a large number of inflammatory factors. The expression of pyroptosis‐associated proteins GSDMD, IL‐1β and IL‐18, especially cleaved subunits, as well as inflammatory mediators including NLRP3, in kidney tissues were examined, together with their localization. The mRNA levels of TNF‐α, IL‐6, IL‐1β and IL‐18 were upregulated in the kidneys by LPS, whereas HBSP significantly blocked the elevation of these cytokines (Figure 2A–D). Both of the precursor and active subunit of GSDMD and IL‐1β, and active IL‐18 protein were significantly increased in the kidneys of LPS‐treated mice in comparison with the control, but they were all decreased by HBSP (Figure 2E–H). In addition, immunofluorescence staining further showed the colocalization of GSDMD‐N, NLRP3, IL‐1β and IL‐18 with AQP1, a key marker of TECs, respectively. The fluorescent intensity of double staining was significantly increased in the LPS group, but decreased by HBSP (Figure 2I).

### 3.3 HBSP reduced apoptosis in kidneys treated with LPS

**FIGURE 3:**
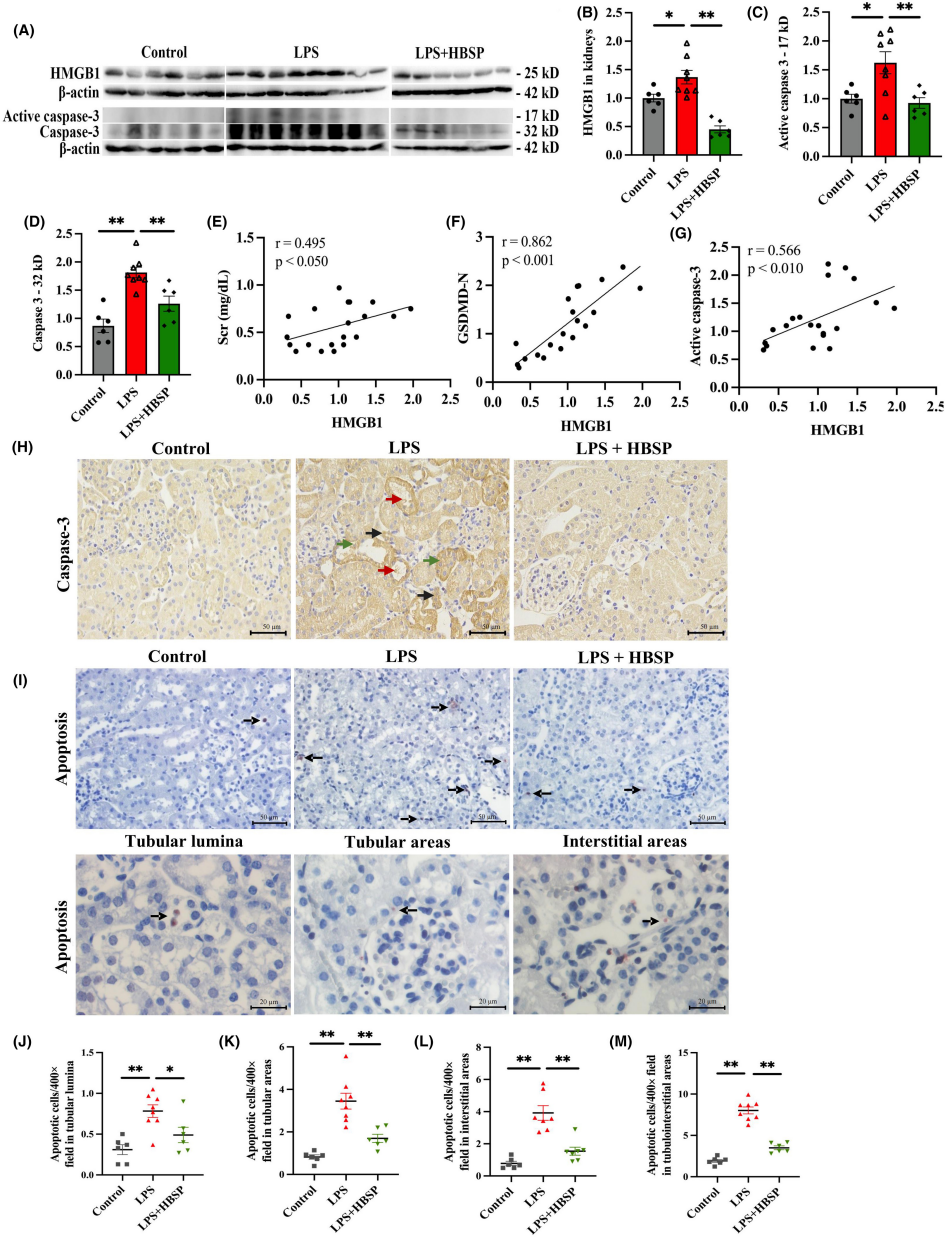
Effects of HBSP treatment on apoptosis in mouse kidneys after LPS treatment. (A–D) Representative western blots of total and active caspase‐3 and HMGB1 and semi‐quantitative analyses. (E–G) Correlations between SCr, GSDMD‐N, active caspase‐3 and HMGB1 respectively. (H) Caspase‐3 staining was visualized in the tubular lumina (red arrow), tubular (blue arrow) and interstitial area (black arrow) of kidneys. Scale bar: 50 μm. (I) Representative photomicrographs showed that positively stained apoptotic cells in different groups, as well as their location in tubular luminal, tubular and interstitial areas, revealed by *in situ* end‐labelling fragmented DNAs. (J–M) The number of apoptotic cells in tubular lumina, tubular, interstitial and tubulointerstitial areas of renal cortex was showed respectively (J‐L) and collectively (M). Scale bar: 50 μm (the upper row) and 20 μm (the lower row). Data were presented as mean ± SEM (*n* = 6–8). *p < 0.05, **p < 0.01.

The cellular apoptosis in the kidney treated with LPS and/or HBSP was evaluated. Compared to the control group, the expression of HMGB1 and cleaved 17 kD caspase‐3 (an executing enzyme of apoptosis) was markedly increased in the kidneys of LPS‐treated mice at 24 h, but significantly decreased by HBSP (Figure 3A–D). The level of HMGB1 was positively correlated with SCr, GSDMD‐N and active caspase‐3 (Figure 3E–G). IHC staining revealed that active 17 kD caspase‐3 was mainly localized in the TECs of the LPS group (Figure 3H). The number of renal apoptotic cells was assessed by *in situ* end labelling fragmented DNAs in kidney tissues. Few apoptotic cells were observed in the cortex of the control group, whereas the number of apoptotic cells in the tubular luminal area, tubular area and interstitial area were all dramatically increased by LPS, and decreased by HBSP (Figure 3I–M).

### 3.4 HBSP reduced F4/80+ macrophage infiltration and promoted M2 polarization in kidneys

**FIGURE 4:**
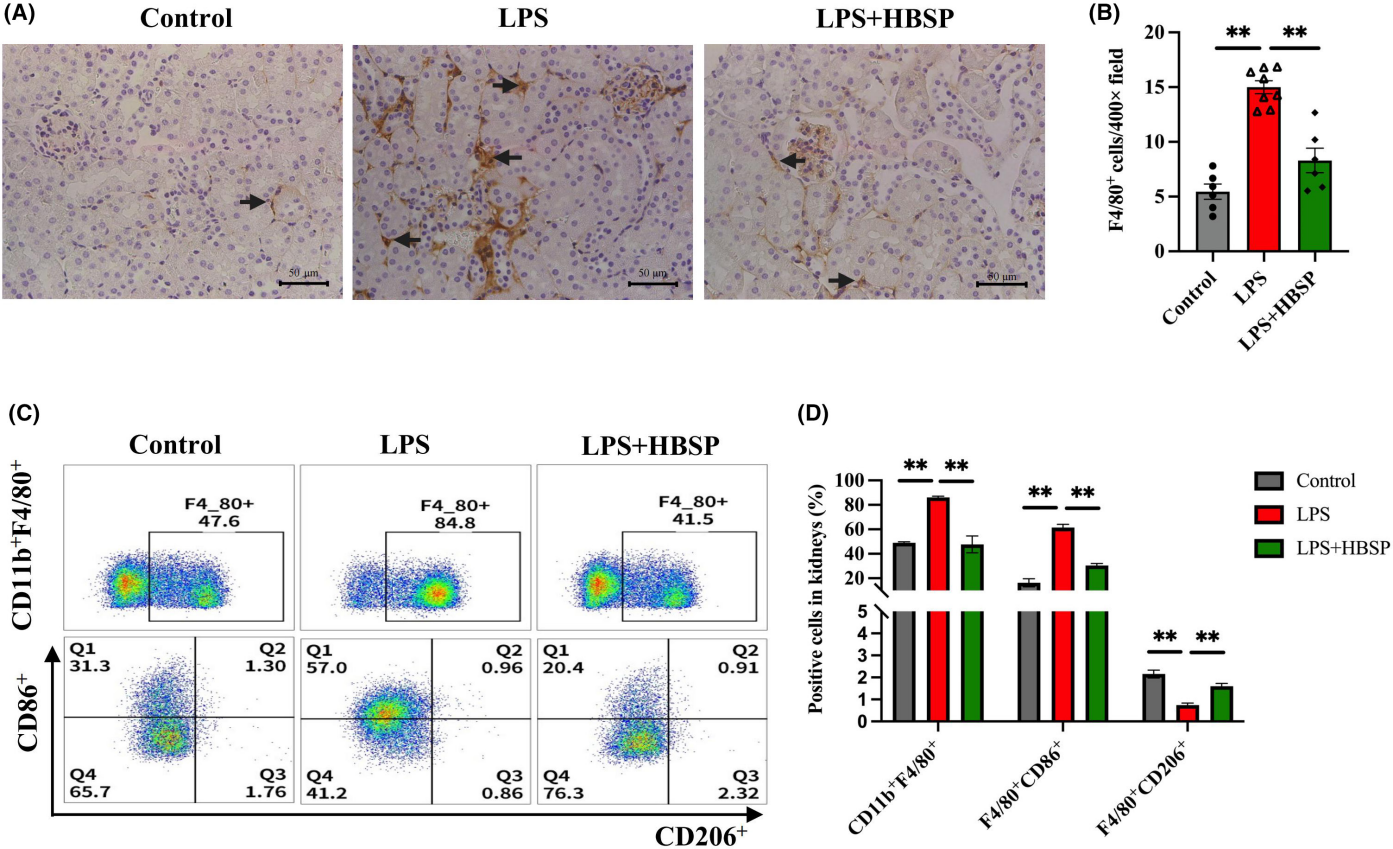
Macrophage infiltration and polarization in LPS‐kidneys with or without HBSP treatment. (A, B) Representative images of F4/80^+^ staining in different kidney compartments (black arrow) and semi‐quantitative analysis (*n* = 6–8). Scale bar: 50 μm. (C, D) The percentage of CD11b^+^F4/80^+^, F4/80^+^CD86^+^ and F4/80^+^CD206^+^ cells in renal tissues was assessed by flow cytometry. Data was presented as mean ± SEM (*n* = 3). **p < 0.01.

Macrophages play key roles in AKI and repair, with the different phenotypes of macrophages exerting pro‐inflammatory or anti‐inflammatory effects. The infiltration and activation of macrophages were detected by IHC staining of F4/80. As shown in Figure 4A,B, F4/80^+^ macrophages in tubulointerstitial areas were remarkably increased by LPS compared to the control group, but decreased by HBSP treatment.

Flow cytometry further revealed that, in contrast to the control group, the percentage of CD11b^+^F4/80^+^ macrophages in kidney tissues was significantly up‐regulated in the LPS group, but decreased by HBSP (Control vs LPS vs LPS + HBSP: 48.93 ± 0.96% vs 86.17 ± 0.91% vs 47.67 ± 6.93%). The selected macrophages were then distinguished using the M1 marker CD86 and the M2 marker CD206. In particular, these changes were dominated by F4/80^+^CD86^+^ M1 macrophages (Control vs LPS vs LPS + HBSP: 16.35 ± 3.28% vs 61.50 ± 2.63% vs 30.43 ± 1.67%). However, the percentage of F4/80^+^CD206^+^ M2 macrophages was decreased by LPS, then increased by HBSP (Control vs LPS vs LPS + HBSP: 2.16 ± 0.18% vs 0.75 ± 0.09% vs 1.60 ± 0.13%, Figure 4C,D).

### 3.5 EPOR expression regulated by LPS and HBSP

**FIGURE 5:**
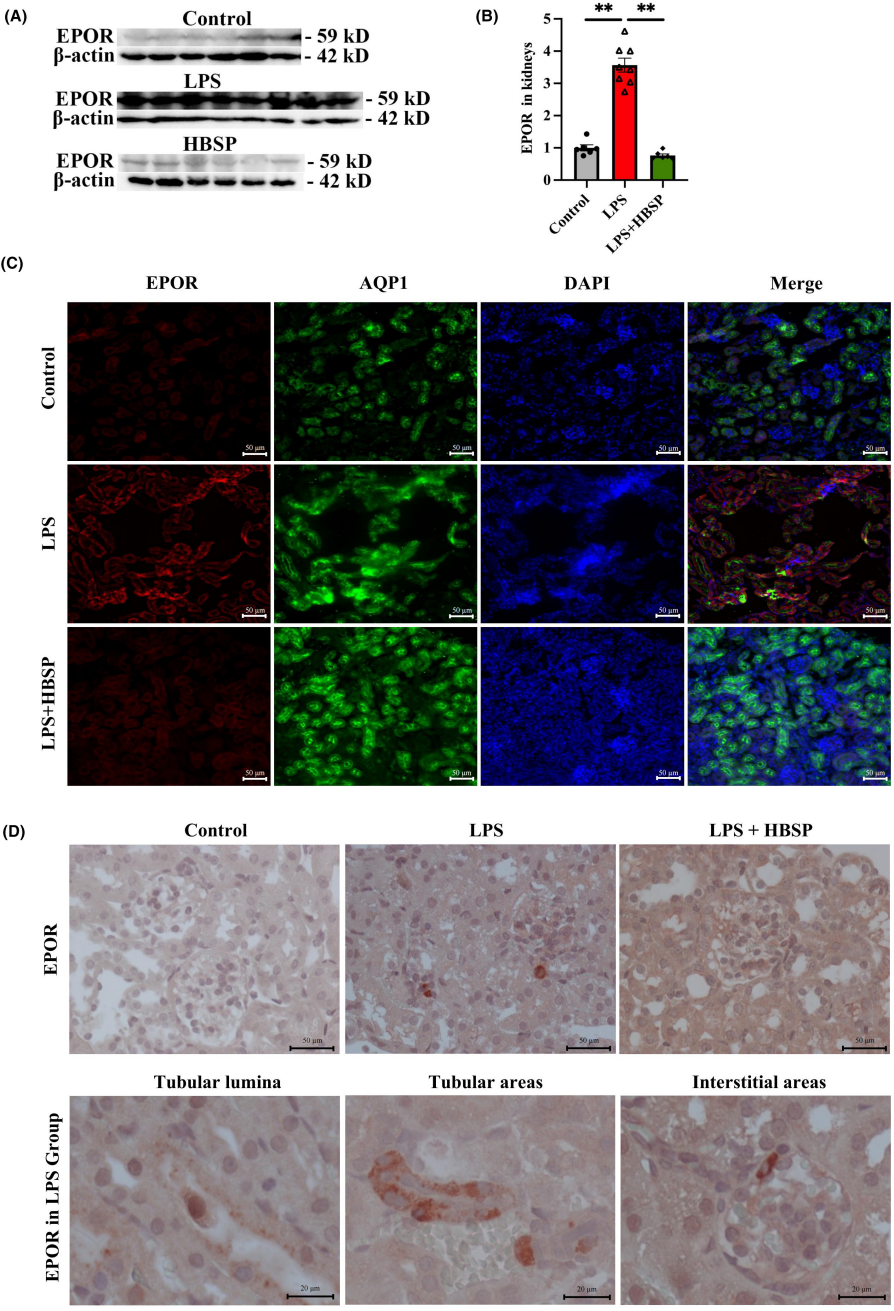
The expression of EPOR in the kidneys treated by LPS and HBSP. (A, B) Representative western blots of EPOR protein and its loading control β‐actin in different groups. Data were presented as mean ± SEM (*n* = 6–8). **p < 0.01. (C) The colocalization of EPOR and AQP1 (a marker of tubular cells) in the cortex of kidneys were detected by immunofluorescence staining (Scale bar: 50 μm). (D) Representative photomicrographs of EPOR protein in different groups, particularly in the LPS group, showed staining in different compartments including tubular lumina, tubular and interstitial areas, as revealed by IHC staining (Scale bar: 50 μm For the upper row and 20 μm for the lower row).

To further understand the change of EPOR modified by LPS and HBSP, the expression and localization of EPOR in kidneys were evaluated. Western blotting demonstrated that the expression of EPOR protein was significantly increased by LPS treatment but downregulated by HBSP (Figure 5A,B). The immunofluorescence staining showed that EPOR protein was intensively distributed in the kidneys of LPS group, but expressed at a very low level in the control group and after HBSP treatment (Figure 5C). EPOR and AQP1 were mainly colocalized in tubular areas of the LPS group. In addition, IHC staining showed that EPOR protein was mainly located in the tubular lumen, the brush border and cytoplasm of TECs in the LPS group, and sometimes in interstitial areas (Figure 5D).

### 3.6 Effects of HBSP on cell viability and inflammation in TCMK‐1 cells treated by LPS

**FIGURE 6:**
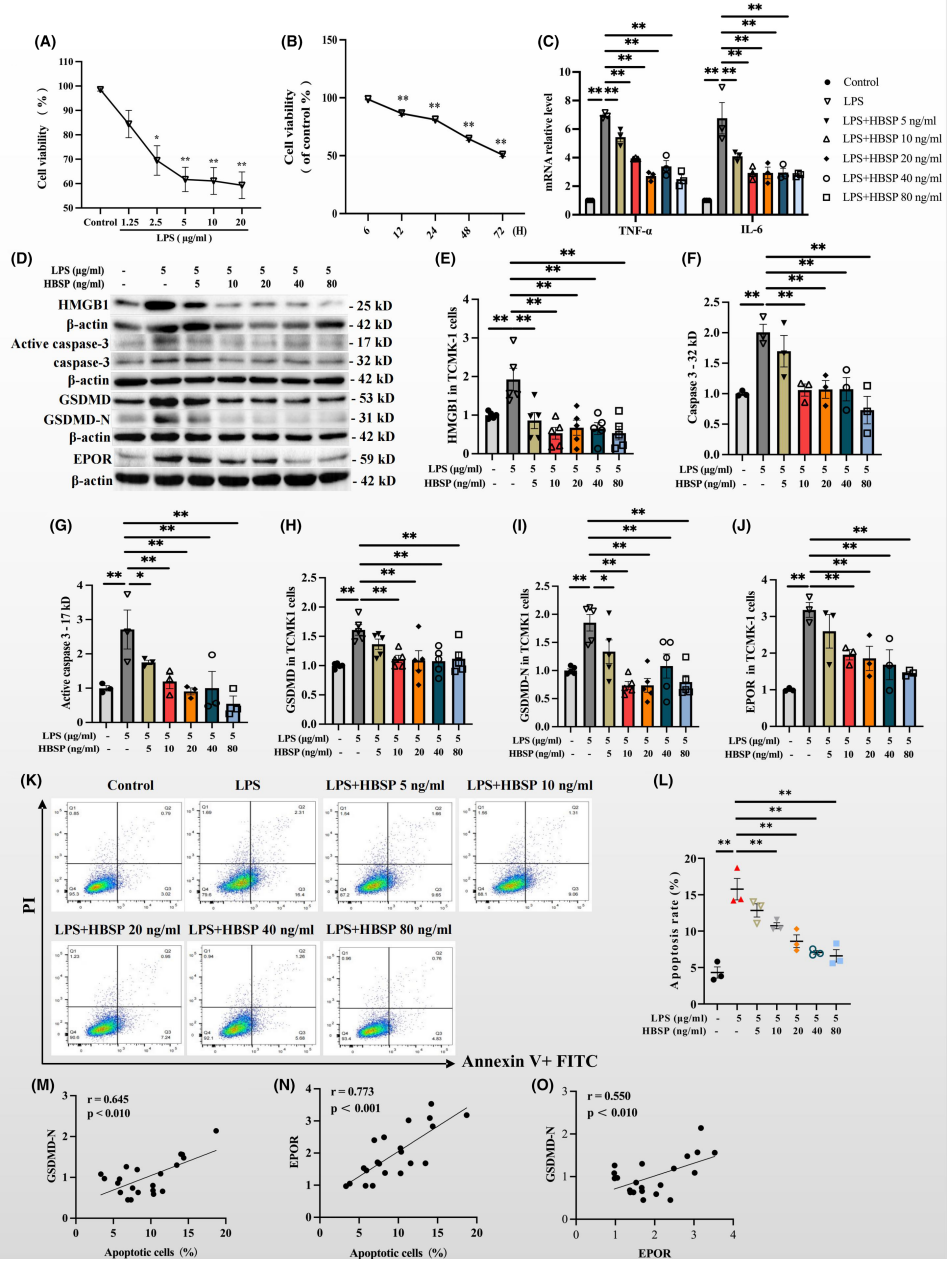
Effects of HBSP on cell viability and death, as well as inflammation upon LPS stimulation in TCMK‐1 cells. (A, B) Cell viability was changed by the LPS of different concentrations (1.25–20 μg/mL) at 24 h, and 5 μg/mL LPS at different time points (0–72 h). (C) The expression of TNF‐α and IL‐6 mRNA was detected by RT‐qPCR in TCMK1 cells treated with 5 μg/mL LPS and/or HBSP (5–80 ng/mL) for 24 h. (D–J) Western blotting analysis of HMGB1, caspase‐3, GSDMD and EPOR in TCMK‐1 cells subjected to LPS and/or HBSP at 5–80 ng/mL for 24 h. (K, L) The percentage of apoptotic cells was detected by flow cytometry (apoptotic cells: AV^+^/AV^+^ and PI^+^; necrotic cells: PI^+^). (M–O) Correlation analysis among apoptotic cells, EPOR and GSDMD‐N. Data were presented as mean ± SEM (*n* = 3–5). *p < 0.05; **p < 0.01; AV, annexin V; PI, propidium iodide.

To mimic AKI *in vitro*, LPS was used to induce cellular injury in a mouse kidney epithelial cell line, TCMK‐1 cells. LPS at 1.25 to 20 μg/mL was used to stimulate TCMK‐1 cells for 24 h. The cell viability detected by CCK‐8 was gradually decreased as the dose of LPS was increased, and this decrease was levelled from 5 to 20 μg/mL (Figure 6A). Then 5 μg/mL LPS was applied to TCMK‐1 cells for 6 to 72 h. The cell viability was decreased time‐dependently (Figure 6B). The expression of TNF‐α and IL‐6 mRNA in TCMK‐1 cells was increased by LPS, but gradually decreased by 5–80 ng/mL HBSP at 24 h (Figure 6C). The expression of apoptosis and pyroptosis‐associated proteins including HMGB1, 32 kD and 17 kD caspase‐3 and GSDMD, as well as EPOR protein, was significantly increased in TCMK‐1 cells subjected to LPS stimulation, whereas HBSP at 5–80 ng/mL significantly decreased the expression of these proteins (Figure 6D–J). To confirmed the above effects of HBSP upon LPS, the number of apoptotic cells was evaluated by AV/PI stanning. The percentage of early and late apoptotic cells was significantly increased by LPS compared to the control group, but was decreased by HBSP dose‐dependently (Figure 6K,L). GSDMD‐N, apoptosis and EPOR were positively corelated with each other respectively (Figure 6M–O).

## 4 DISCUSSION

**FIGURE 7:**
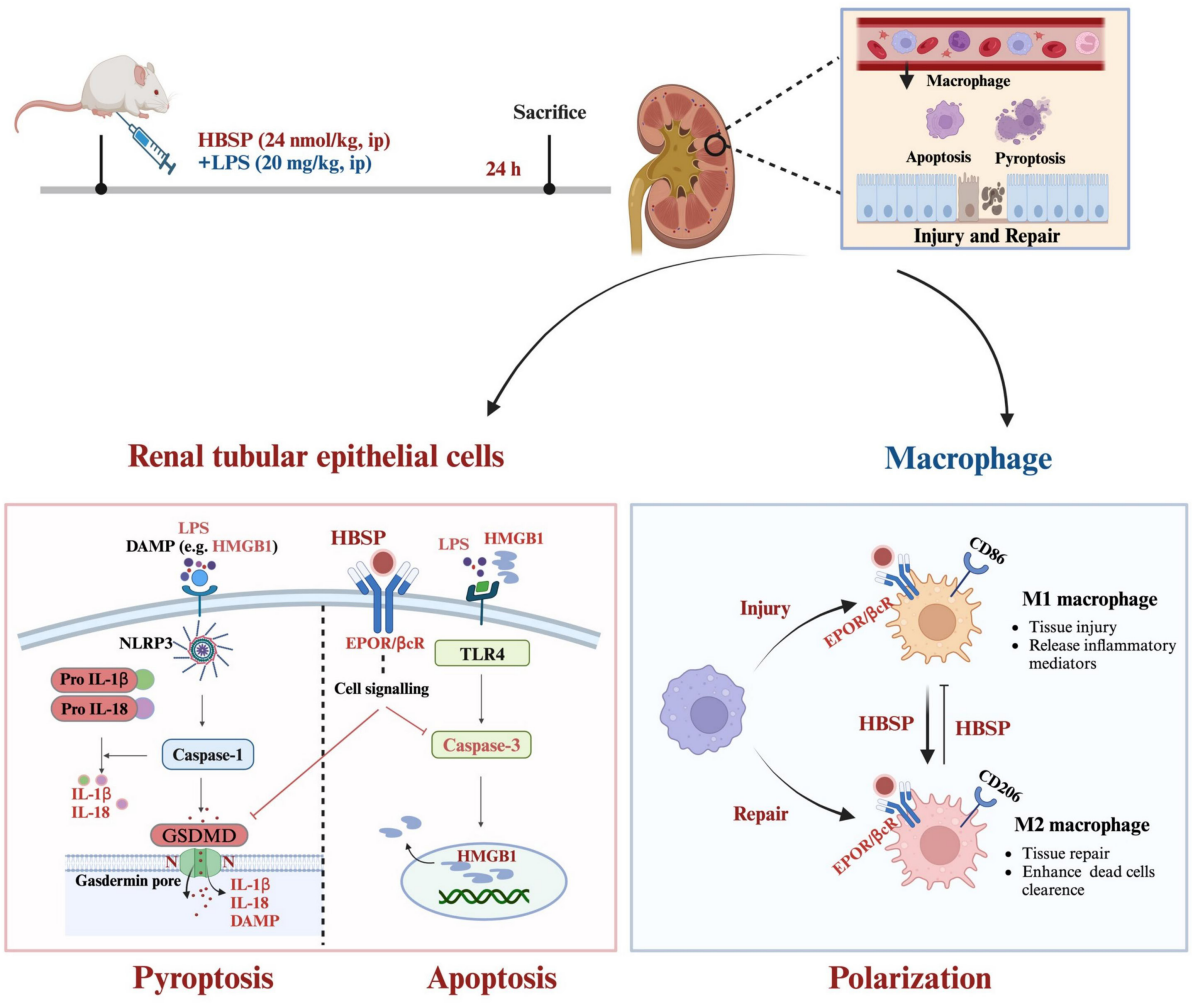
Schematic summary of mouse experimental design for studying the effect of HBSP in LPS‐induced AKI. LPS was injected intraperitoneally to mice with or without HBSP at the same time for 24 h. LPS activates NLRP3 inflammasomes and cleavages GSDMD leading to caspase‐1 dependent pyroptosis and releasing pro‐inflammatory cytokines. Additionally, LPS triggers caspase‐3 activation and apoptosis. LPS also induces macrophage infiltration and polarization to M1 phenotype. HMGB1, one of DAMPs, participates in the multiple forms of cell death, including apoptosis and pyroptosis. EPOR is expressed on the surface of TECs and macrophages. HBSP only binds to innate repair receptor (EPOR/βcR) and exerts protection. HBSP treatment mitigates both pyroptosis and apoptosis, reduces inflammation, and promotes macrophage M2 polarization to facilitate kidney repair. DAMP, damage‐associated molecular pattern; EPOR, erythropoietin receptor; HBSP, helix B surface peptide; HMGB1, high‐mobility group box 1; LPS, liposaccharide; TEC, tubular epithelial cell; βcR, β common receptor. (Created in BioRender. Huang, L. (2024) BioRender.com/f82d381).

HBSP, in this study, significantly preserved renal function and structure in LPS‐induced AKI by reducing pyroptosis and apoptosis mainly in TECs, as well as improved systemic inflammation. In both LPS‐treated mouse kidneys and cultured tubular epithelial cells, HBSP markedly reduced the expression of pyroptosis and apoptosis‐associated proteins including GSDMD‐N and the cleaved subunits of IL‐1β, IL‐18 and caspase‐3. In addition, HBSP promoted the phenotypic transformation of macrophages from M1 to M2. Therefore, this study provides mechanistic insights into the renoprotection of HBSP against LPS‐AKI, in terms of regulating multiple cell death modes and immune responses (Figure 7). Our previous studies have shown that HBSP and cyclic form of HBSP (CHBP) mitigated kidney apoptosis, inflammation and oxidative stress, but enhanced TECs phagocytosis to promote kidney recovery post IR injury.^24^, ^28^, ^29^, ^30^ The protective effects were also induced by CHBP on decreasing the formation of NLRP3 inflammasome and the secretion of IL‐1β via the NF‐κB pathway in LPS‐induced acute lung injury.^31^ HBSP reduces sepsis‐induced kidney injury via PI3K/Akt Pathway.^26^ Indeed, regulated cell death including apoptosis and necrosis, in terms of pyroptosis, necroptosis and ferroptosis, have emerged as central events in the pathogenesis of AKI, which may be amenable by therapeutic intervention. The different forms of cell death cannot exist in isolation, but share triggers, molecular components and protective mechanisms and potentially transdifferentiated or coexisted.^32^ However, it was still unclear how HBSP regulated the various forms of programmed TEC death in SA‐AKI. In this study, it has been demonstrated that LPS induced systemic and renal inflammation, and the expression of key pyroptotic and apoptotic proteins in both *in vivo* and *in vitro* models. Moreover, the co‐localization of immunofluorescent dyes (labelling cell death and cell type markers) was further demonstrated that TECs were the major cells underwent pyroptosis and apoptosis. HBSP also greatly ameliorated systemic inflammation and preserved kidney structure and function, which was consistent with the finding of others.^26^ However, we demonstrated that HBSP decreased not only renal cell apoptosis by reducing active caspase‐3, but also pyroptosis associated proteins including GSDMD‐N and the cleaved subunits of IL‐1β and IL‐18. These findings expand our knowledges regarding the protection of HBSP against LPS‐AKI.

DAMPs, in addition, released by excessive damaged parenchymal cells also contributed to persistent inflammation that leads to secondary tissue injury.^33^ HMGB1 is a critical DAMP that plays key roles in both apoptosis and pyroptosis in SA‐AKI.^34^, ^35^ The high serum level of HMGB1 was associated with the adverse outcomes of septic patients.^36^ Our study revealed that HMGB1 expression was significantly upregulated by LPS stimulation, but downregulated by further HBSP treatment. Correlative analysis revealed that HMGB1 was positively correlated with SCr, GSDMD‐N and cleaved caspase‐3, showing its association with renal function, pyroptosis and apoptosis. These findings disclose the mechanism of HBSP in regulating inflammatory and cell death in LPS‐induced AKI, and facilitate its potential clinical applications.

Macrophages actively participate in the pathological processes of AKI and are significantly associated with its outcome.^37^ Massive macrophages infiltration was showed in renal biopsies from sepsis patients,^38^ but depletion of M2 macrophages exacerbated renal damage and delayed kidney repair at the late stage of sepsis in animal models.^39^ Drugs reducing macrophage infiltration and inducing macrophage polarization have been proven to be effective against SA‐AKI.^40^, ^41^ LPS recognizes toll‐like receptors on macrophages and upregulates inflammatory mediators, which lead to the M1 polarization of macrophages and aggravated tissue injury.^42^ The high‐dosage of EPO suppressed macrophage M1 polarization with reduced iNOS expression and alleviated systemic inflammation, showing the protective effects on SA‐AKI patients,^43^ whereas side‐effects were also induced, such as hypertension and thrombosis, but could be avoided by using HBSP, only binds to EPOR/βcR.^44^ This study showed significant macrophage infiltration in the kidneys following LPS‐stimulation, with a predominance of M1 phenotype characterized by heightened CD68 expression. HBSP treatment alleviated macrophage infiltration, promoted macrophages transformation to M2 phenotype that highly expresses CD206. This study indicates that the renoprotective role of HBSP against LPS‐AKI attributed to HBSP specifically bound to EPOR/βcR on macrophages, thereby inhibiting macrophage infiltration and inflammatory responses, and directly promoting macrophage M2 polarization.

EPOR remains low expression in normal kidneys, but was quickly upregulated under stress of IR injury.^45^ However, persistent high expression of renal EPOR resulted in aggravated renal fibrosis at the chronic stage of kidney IR injury.^46^ It is implying that EPOR is not only a damage indicator, but also an important repair or fibrotic marker. EPOR expression was significantly increased in IR‐kidneys, but decreased in HBSP treatment.^21^ Similarly, the expression of EPOR was also increased in the kidneys treated by LPS, which was decreased by HBSP treatment. EPOR, in this study, is positively correlated with the pathophysiological changes including inflammation, apoptosis and pyroptosis in the kidneys. After LPS stimulation, renal tubular epithelial cells experience exacerbated inflammation and elevated levels of apoptosis and pyroptosis, while at the same time initiated the process of repair including increased EPOR expression. Conversely, after HBSP treatment, the level of inflammation in the kidneys was decreased, while EPOR expression was also declined, and the rates of apoptosis and pyroptosis were decreased. Therefore, the change of EPOR expression reflects, to some extent, the state of cellular injury, as well as recovery. The analyses of correlations encompassed all changes between the LPS‐induced injury, HBSP‐promoted repair and EPOR involvement. In addition, EPOR expression may also regulate the interaction between macrophages and TECs. Taken together, LPS induced apoptosis and pyroptosis, which could stimulate the expression of EPOR that further activated phagocytes including macrophage to clear dead cells, limit further inflammatory responses.

This study presents promising evidences for the potential clinical application of HBSP on SA‐AKI. Future studies are needed to assess the long‐term effects of early HBSP administration on LPS‐induced AKI. It is also crucial to delineate the mechanistic signalling pathways of HBSP renoprotection, in particular, in‐depth interplays between pyroptosis, apoptosis, inflammation, repair and fibrosis. Furthermore, the activation of EPO/EPOR signalling pathway could promote macrophages to find dying cells and enhance macrophage phagocytosis of death cells, and immune tolerance.^47^ It is worth to investigate how EPOR or EPO/EPOR influences macrophage polarization, and the phagocytic activity of different macrophages, the interaction of TECs and macrophages, as well as the change of EPOR/βcR in comparison to EPOR, and their associations with the outcome of LPS‐AKI.

HBSP not only plays a protective role in the kidney function and histology by reducing apoptosis, but also effectively decreased pyroptosis, which subsequently limited inflammation and promoted macrophage M2 polarization. Moreover, HBSP also limited the elevation of EPOR, which might be associated with potential fibrosis.

## AUTHOR CONTRIBUTIONS

**Lili Huang:** Data curation (equal); formal analysis (equal); funding acquisition (equal); software (equal); writing – original draft (lead). **Yuanyuan Wu:** Conceptualization (equal); data curation (equal); formal analysis (equal); writing – review and editing (equal). **Wenli Sai:** Resources (equal). **Yanan Wang:** Methodology (equal); validation (lead). **Guijuan Feng:** Methodology (equal); software (equal). **Yuqing Lu:** Methodology (equal); software (equal). **Fei Chen:** Methodology (equal). **Xinzhong Huang:** Supervision (equal). **Hongsheng Zhao:** Supervision (equal); writing – review and editing (equal). **Zhifeng Gu:** Funding acquisition (supporting); supervision (equal); writing – review and editing (equal). **Bin Yang:** Conceptualization (lead); funding acquisition (lead); project administration (lead); supervision (lead); writing – review and editing (lead).

## FUNDING INFORMATION

This research was funded by National Natural Science Foundation of China (grant number 81873622 to Bin Yang, 82200765 to Yuanyuan Wu), Jiangsu Provincial Medical Key Discipline Cultivation Unit (grant number JSDW202205 to Zhifeng Gu), Postgraduate Research & Practice Innovation Program of Jiangsu Province (grant number KYCX20_2804 to Lili Huang) and Nantong Science and Technology Foundation (grant number MS22022092 to Lili Huang).

## CONFLICT OF INTEREST STATEMENT

The authors declare no conflicts of interest.

## Data Availability

The data that support the findings of this study are available on request from the corresponding author.
